# Metabolic programming in tooth development: a regulatory network from energy substrates to signaling instructions

**DOI:** 10.3389/fcell.2026.1779410

**Published:** 2026-03-06

**Authors:** Xiaoyu Cao, Yiping Gao, Wen Liu, Xiaojun Sun

**Affiliations:** 1 Department of Oral Medicine, First Hospital of Shanxi Medical University, Taiyuan, Shanxi, China; 2 Shanxi Medical University and Hospital of Stomatology, Taiyuan, Shanxi, China

**Keywords:** tooth development, metabolomics, metabolic reprogramming, metabolic signaling, epigenetic regulation

## Abstract

Tooth development is a process meticulously orchestrated by complex signaling networks. Traditionally, metabolism has been viewed as a passive supplier of energy and building blocks. This review, by systematically integrating recent evidence, proposes that metabolism acts as an active programmer during tooth development, whose functions extend beyond fundamental support. We elaborate a dynamic metabolic regulatory framework wherein cellular metabolic status engages in deep, bidirectional crosstalk with classic developmental pathways such as Wnt, BMP, FGF, and Hh through four core mechanisms: energy sensing and fate decision, moonlighting signaling functions of metabolic enzymes, metabolite-mediated epigenetic remodeling, and specific substrate metabolism. This crosstalk guides cell behavior, tooth morphogenesis, and matrix mineralization in a spatiotemporally specific manner. Evidence from models of hereditary or acquired metabolic disorders strongly underscores the physiological and pathological relevance of this network. Building on this integrative perspective, we further discuss how emerging technologies—including spatial multi-omics, organoids, and computational modeling—can deepen mechanistic understanding, and explore the translational potential of targeting metabolic nodes for early diagnosis, prevention, and regenerative therapy. This review aims to provide a systematic discussion on the central regulatory role of metabolic status in tooth development, with a focus on the metabolism-signaling integrative network, thereby offering a more comprehensive conceptual framework for elucidating developmental principles, disease mechanisms, and informing novel strategies in oral medicine.

## 1 Introduction

Metabolomics, a crucial branch of systems biology, focuses on the qualitative and quantitative analysis of small molecule metabolites within living organisms (Mosteiro et al., 2021). These metabolites—which may be metabolic intermediates or products of gene expression and enzyme activity—represent downstream outcomes of genomic, transcriptomic, and proteomic activities while also participating in complex biological networks through feedback regulation (Ahmad et al., 2025). Compared with genomics and proteomics, metabolomics directly captures terminal information of biological systems in specific spatiotemporal contexts, thereby more accurately reflecting physiological and pathological processes that have already occurred *in vivo*, and serving as a critical bridge linking molecular mechanisms to clinical phenotypes.

Tooth development is a highly conserved and exceptionally intricate morphogenetic process, requiring the precise spatiotemporal coordination of epithelial-mesenchymal crosstalk, meticulous cell fate determination, and extracellular matrix biomineralization, culminating in the formation of uniquely shaped and functionally specialized mineralized tissues (Kollar and Baird, 1969). For nearly a century, research in this field has primarily focused on a set of conserved signaling pathways, such as bone morphogenetic protein (BMP), fibroblast growth factor (FGF), Hedgehog (Hh), and Wnt pathways, which constitute a classic regulatory paradigm: specific signaling centers established in the epithelium transmit patterning information to the mesenchyme via ligand–receptor interactions, thereby driving cell proliferation, fate specification, and tissue morphogenesis (Thesleff, 2003). This classical “signaling center–responding tissue” framework has profoundly shaped and, to some extent, constrained our understanding of the program underlying tooth development. Particularly, it primarily maps the “wiring diagram” of signal transduction, leaving the “fundamental drivers” for executing this blueprint and the basis for cellular perception and decision-making during fate choices insufficiently explained.

The very capability of metabolomics to capture terminal information prompts a re-evaluation of the role of metabolism in development. In recent years, with the rapid advancement in fields such as metabolomics, epigenetics, and cellular energy biology, cellular metabolism—once regarded merely as a “logistical support system” providing energy and biosynthetic precursors—is being redefined as a central regulator of life processes (Shyh-Chang et al., 2013a). In developmental biology, metabolic reprogramming has been demonstrated to play a decisive role in maintaining embryonic stem cell pluripotency, somatic cell reprogramming, and the development of various organs (Zhang et al., 2018). This paradigm shift prompts us to re-examine tooth development: metabolic activity is not merely a consequence of morphogenesis but may well be the upstream cause directing its orderly progression.

Growing evidence supports this new perspective. Studies have shown that the metabolic states of odontogenic cells (including both epithelial and mesenchymal cells) are not merely passive followers but active participants and directors of the developmental process (Zhang et al., 2025). Through four interconnected core mechanisms—providing energy (Yan et al., 2021), supplying biosynthetic precursors (Yoshida et al., 2024), acting as signaling molecules (Yang et al., 2011), and shaping the epigenetic landscape (Verdikt and Allard, 2021)—the metabolic network constitutes a dynamic and instructive programming system in four dimensions. This system not only provides the material foundation for tooth development but also deeply regulates key events such as cell fate determination, tissue patterning, and extracellular matrix mineralization. Therefore, understanding metabolic programming is indispensable for unraveling the precise regulatory mysteries of tooth morphogenesis.

In this review, we aim to propose and elucidate the emerging framework of metabolic programming in tooth development, arguing that metabolic activity serves as an instructive signal driving morphogenesis, rather than merely energetic support. By doing so, we hope to shift the research perspective in this field from focusing on linear signaling pathways toward understanding a dynamic, integrated metabolic regulatory network, thereby laying a new theoretical foundation for revealing the fundamental principles of tooth development, the etiology of related diseases, and exploring novel regenerative strategies.

## 2 Four core mechanisms of metabolic regulation in tooth development: the molecular logic from energy sensing to matrix construction

The classic signaling pathways in tooth development—Wnt, BMP, Shh, and FGF—do not operate in a metabolic vacuum. On the contrary, the metabolic state of cells forms a fundamental regulatory layer that governs the activation, perception, and execution of these pathways. Through the following four mechanisms, metabolism directly determines the efficacy and specificity of signaling pathways, thereby transforming nutritional and energetic information into precise morphogenetic instructions.

### 2.1 The core regulatory network of cellular energy metabolism: from ATP supply to cell fate determination

Every step of tooth development relies on sustained and adequate energy supply. Cellular energy metabolism does not only respond to demand, but also actively shapes key processes such as cell proliferation, differentiation, and functional execution through a multi-layered network that integrates ATP generation, energy sensing, and signal transduction.

Mitochondrial Oxidative Phosphorylation: The Power Core for Biosynthesis and Secretion. During tooth development, especially in the hard tissue formation stage, cellular activities are highly energy-intensive. Processes such as the synthesis and secretion of large amounts of enamel matrix proteins by ameloblasts, and the active collagen synthesis and ion transport by odontoblasts are all highly dependent on efficient mitochondrial oxidative phosphorylation (OXPHOS) to provide substantial ATP (Yan et al., 2021). Research has demonstrated that in mice with specific knockout of mitochondrial function in dental epithelial cells, ameloblasts completely lose their ability to secrete enamel proteins, resulting in a failure of enamel formation (Imhof et al., 2020). This directly proves that mitochondrial OXPHOS serves as an irreplaceable energetic cornerstone supporting terminal secretory functions.

AMPK/mTOR: The Upstream Hub for Sensing Energy Status and Guiding Cellular Behavior. How do cells make the decision to “grow” or “differentiate” based on the availability of energy and nutrients? The AMP-activated protein kinase (AMPK) and the mechanistic target of rapamycin (mTOR) form a pair of core energy-sensing and integration hubs that monitor intracellular energy (ATP/AMP ratio) and nutrient signals (amino acids, glucose) (Yi et al., 2021). In the early stages of tooth germ development, rapidly proliferating cells require extensive biosynthesis. At this stage, the mTORC1 complex is activated, promoting protein and lipid synthesis while inhibiting autophagy, thereby driving tooth germ growth (Saxton and Sabatini, 2017). However, when cells initiate differentiation programs (e.g., odontoblast differentiation), their energy demand shifts from “biosynthesis” to “specialized functional execution” (e.g., massive collagen secretion). At this point, AMPK is activated. It phosphorylates Raptor to inhibit mTORC1 activity, thereby shutting down energy-consuming anabolic processes, initiating autophagy to recycle intracellular components, and upregulating genes related to mitochondrial biogenesis to provide efficient oxidative phosphorylation support for differentiation (Herzig and Shaw, 2018). Studies have shown that glucose and serum deprivation in stem cells from human exfoliated deciduous teeth (SHED) strongly activated AMPK, which led to a significant decrease in cell proliferation, cell cycle arrest at the S phase, and increased apoptosis. More importantly, the sustained activation of AMPK altered the lineage commitment of the stem cells, manifesting as enhanced chondrogenic differentiation potential and reduced osteogenic differentiation capacity (Pawar et al., 2021). This mechanism ensures that cells engage in constructive growth only when energy is sufficient, and switch to differentiation pathways when energy is relatively limited or functional specialization is required, making it a crucial switch for resource allocation and cell fate determination.

Deep Integration of Metabolic Signals with Classic Developmental Pathways. The AMPK/mTOR hub not only directly regulates metabolism but also engages in deeper crosstalk with the classic signaling pathways that guide tooth development, forming a sophisticated regulatory network (Hardie et al., 2012). For instance, there is significant functional integration with the Wnt pathway. Under energy stress, AMPK can phosphorylate the TSC2 protein, which creates a condition for glycogen synthase kinase 3 (GSK3) to further phosphorylate TSC2. This collaborative action activates TSC2 and subsequently inhibits mTOR activity (Inoki et al., 2006). Crucially, activated Wnt signaling can inhibit GSK3 activity, thereby blocking the aforementioned phosphorylation cascade that suppresses mTOR. This ultimately leads to the activation of the mTORC1 signaling pathway (Choo et al., 2006). This discovery reveals that, beyond regulating gene transcription, Wnt signaling can also directly promote protein synthesis and cell growth by relieving the inhibition on mTOR. AMPK and mTOR signaling also play key regulatory roles in the BMP pathway. In bone and tooth development, the activation of AMPK enhances BMP-Smad signal transduction, promotes the expression of osteogenic/odontogenic-related genes, and drives cell differentiation and matrix mineralization. Conversely, activated mTORC1 signaling exerts negative feedback inhibition on the BMP pathway, interfering with Smad protein function to prevent premature differentiation (Rahman et al., 2015). During tooth development, this crosstalk among AMPK, mTOR, Wnt, and BMP signaling likely provides critical control nodes for coordinating the proliferation and differentiation of odontogenic cells.

### 2.2 Specific substrate metabolism: constructing and modulating the signaling molecule microenvironment

The formation of dental hard tissues (enamel and dentin) is critically dependent on specific substrate metabolic pathways to synthesize matrix proteins and polysaccharides with correct post-translational modifications. Among these, sulfate metabolism serves as a paradigmatic example linking cellular metabolism to extracellular matrix (ECM) assembly and mineralization.

Sulfated glycosaminoglycans, notably chondroitin sulfate and heparan sulfate, and proteoglycans in the ECM act as scaffolds mediating cell adhesion, growth factor presentation, and mineralization nodule formation (Hayes and Melrose, 2023; Soares da Costa et al., 2017). Sulfate is the sole sulfur donor for the sulfation modification of these molecules. The Slc26a2 (sulfate transporter) located on the membranes of odontoblasts and ameloblasts is responsible for the active transport of inorganic sulfate from plasma into cells. Subsequently, sulfate is activated into the high-energy carrier phosphoadenosine phosphosulfate (PAPS) by ATP sulfurylase (ATPS) and PAPS synthase. This activation process consumes substantial ATP, once again tightly coupling matrix synthesis with the cellular energy state (Zarinfar et al., 2025).

Animal model studies have definitively confirmed the indispensability of sulfate metabolism. Slc26a2 gene knockout mice exhibit severe systemic chondrodysplasia, and SLC26A2 variants are associated with various clinical manifestations in the craniofacial region, including increased upper facial height, micrognathia, high-arched palate, cleft palate (25%–60%), tooth agenesis (30%), and microdontia (Härkönen et al., 2021). The dental phenotype is particularly characteristic: tooth germ development arrests at the early bell stage, odontoblast differentiation is abnormal, the predentin zone widens with severely impaired mineralization, and the enamel epithelium is disorganized, failing to form normal rod structures (Yoshida et al., 2024).

Mechanistically, interrupted sulfate supply likely leads to impaired synthesis of sulfated glycosaminoglycans. This alters the composition of the ECM in the dental papilla and follicle, weakening its ability to bind and sequester morphogens like BMP and FGF, thereby disrupting the concentration gradient required to induce odontoblast differentiation (Inubushi et al., 2024); Secondly, heparan sulfate proteoglycans are essential co-factors for the assembly and stabilization of BMP receptor complexes on the cell membrane. Their undersulfation directly causes a significant decrease in BMP-Smad signaling efficiency (Nadanaka and Kitagawa, 2008; Le Pennec et al., 2024); Furthermore, heparan sulfate proteoglycans are also implicated in regulating canonical Wnt signaling during tooth development (Inubushi et al., 2023). These constitute the core molecular mechanisms leading to differentiation arrest; Finally, the sulfation of phosphoproteins in the dentin matrix is crucial for the nucleation, growth, and orientation of hydroxyapatite crystals. Sulfate deficiency deprives the matrix of its normal ability to guide mineralization (Hayano et al., 2012).

Sulfate metabolism does not operate in isolation. It interfaces with cysteine metabolism, jointly maintaining cellular redox balance and sulfur homeostasis (Pajares, 2025). Moreover, sulfate activation depends on ATP provided by mitochondrial oxidative phosphorylation, while the synthesis of sulfated proteoglycans requires UDP-sugar substrates supplied by carbohydrate metabolism (Inubushi et al., 2024). Therefore, sulfate metabolism serves as an integrative network hub for energy, amino acid, and carbohydrate metabolism during tooth development, such that its dysfunction triggers systemic developmental defects.

### 2.3 Non-classical functions of metabolic enzymes: the “moonlighting” signaling roles of PKM2 and ACLY

Specific metabolic enzymes possess “moonlighting” capabilities, shuttling between metabolic catalysis and gene transcriptional regulation, serving as core carriers through which metabolism directly controls development. Among these, the roles of the pyruvate kinase M2 isoform (PKM2) and ATP-citrate lyase (ACLY) are particularly prominent.

In proliferation-active regions of the tooth germ, such as the dental mesenchyme during the bud stage, PKM2 primarily exists as a dimer. Its low catalytic activity favors the accumulation of glycolytic intermediates for biosynthesis. More importantly, upon stimulation by growth factors FGF2, PKM2 can be specifically recruited to the nucleus, where it performs crucial “moonlighting” transcriptional regulatory functions (Yang et al., 2011). Within the nucleus, PKM2 regulates gene expression through at least two mechanisms: First, acting as a protein kinase, it directly phosphorylates histone H3 at threonine 11 (H3T11). This modification is closely associated with the transcriptional activation of cell cycle genes such as Cyclin D1 and c-Myc, thereby maintaining the proliferative potential of the cells (Yang et al., 2014; Venneti and Thompson, 2013). Second, PKM2 can function as a transcriptional co-activator by forming a complex with β-catenin, a key effector of the Wnt pathway, and enhancing its binding to target gene promoters. During tooth development, this complex has been shown to co-activate canonical Wnt target genes like Axin2 and Lef1, which are crucial for maintaining the stemness of dental mesenchymal cells and inhibiting their premature differentiation (Rihan et al., 2025). Furthermore, recent studies indicate that PKM2 can also interact with Gli1, a transcription factor of the Hedgehog pathway. During cusp morphogenesis, they cooperatively promote the transcription of Ptch1 and Gli1 itself, forming a positive feedback loop (Xu et al., 2014). This directly couples the high glycolytic metabolic state with the pro-proliferative Hedgehog signaling pathway, influencing the initiation and patterning of tooth cusps (Zhang et al., 2019a). Therefore, PKM2 serves as a critical molecular node linking cellular metabolic status to the core transcriptional programs of tooth morphogenesis.

ACLY is a core enzyme that connects mitochondrial metabolism to nuclear epigenetic programming. It converts citrate produced by the mitochondrial tricarboxylic acid (TCA) cycle into cytosolic acetyl-CoA. Acetyl-CoA serves not only as a precursor for fatty acid synthesis but also as the exclusive acetyl donor for histone acetyltransferases (p300/CBP) (Murthy et al., 2024). This spatiotemporally specific metabolic remodeling directly drives chromatin opening at enamel protein gene loci. Research indicates that acetyl-CoA generated via ACLY is specifically utilized to catalyze the acetylation of histone H3 at lysine 27 (H3K27ac) in the promoter regions of key ameloblast genes, which is an active transcription enhancer mark (Lazaropoulos et al., 2024). In research models of mineralized tissue formation, modulating acetyl-CoA availability by regulating ACLY has been shown to globally alter the histone acetylation landscape, thereby affecting the expression programs of osteogenic/odontogenic genes (Guo et al., 2021). During ameloblast differentiation, upregulated ACLY activity leads to increased local nuclear Acetyl-CoA concentration. This drives hyperacetylation of histones in the promoter regions of enamel protein genes like AMELX, opening the chromatin structure and facilitating the burst of transcription of these specific genes to meet the high demand for enamel matrix protein synthesis (Wellen et al., 2009).

### 2.4 Metabolites as donors for epigenetic modifications: reshaping the chromatin environment of signaling pathways

Metabolites serve as substrates or cofactors for epigenetic modifying enzymes, globally or locally regulating the expression of target genes for all signaling pathways by altering chromatin accessibility.

Lactate: Long regarded merely as an end-product of anaerobic glycolysis. recent studies have revealed its signaling functions. Lactate induces histone lactylation, a novel epigenetic mark (Meng et al., 2025), directly activating genes related to dentin and enamel matrix synthesis and mineralization (Liu et al., 2023; Yang et al., 2025). This unveils that end-products of carbohydrate metabolism can also directly participate in the epigenetic programming of cell differentiation.

Acetyl-CoA and Histone Acetylation: Acetyl-CoA is not only a key intermediate at the intersection of carbohydrate, lipid, and amino acid metabolism but also the sole acetyl group donor for histone acetyltransferases (HATs) catalyzing histone acetylation reactions (Bradshaw, 2021). Dynamic changes in the nuclear concentration of acetyl-CoA within specific cells (e.g., ameloblasts) can directly regulate the acetylation levels of core histones (such as H3K9, H3K27) (Zhou et al., 2019). During tooth development, this likely directly determines the chromatin accessibility and transcriptional activity of key differentiation genes, such as those encoding enamel proteins and dentin matrix proteins.

The α-KG/Succinate Ratio and Histone/DNA Demethylation: α-Ketoglutarate (α-KG) is an essential cofactor for TET family DNA demethylases and JMJD family histone demethylases, while succinate acts as a competitive inhibitor. The dynamic intracellular balance between α-KG and succinate directly determines the methylation status at gene loci related to tooth development, thereby setting the epigenetic threshold for signaling pathway activity (Carey et al., 2015). During tooth germ development, maintaining a balanced ratio of α-KG to succinate is crucial. α-KG promotes H3K9/H3K27 demethylation reactions (Carey et al., 2015), potentially activating the expression of key odontogenic genes including DSPP and AMELX. Conversely, succinate, as a competitive inhibitor of these enzymes, suppresses the demethylation process (Zhu et al., 2022), leading to the silencing of tooth development-related genes.

S-Adenosylmethionine (SAM): As the primary methyl group donor, the level of SAM directly dictates the global levels of histone and DNA methylation. Disruption in methionine metabolism leading to SAM depletion causes genome-wide hypomethylation (Shyh-Chang et al., 2013b), which has been shown to affect the stemness and differentiation capacity of dental pulp stem cells (Yang et al., 2021).

### 2.5 Integration and interaction of the metabolic regulatory network

**FIGURE 1:**
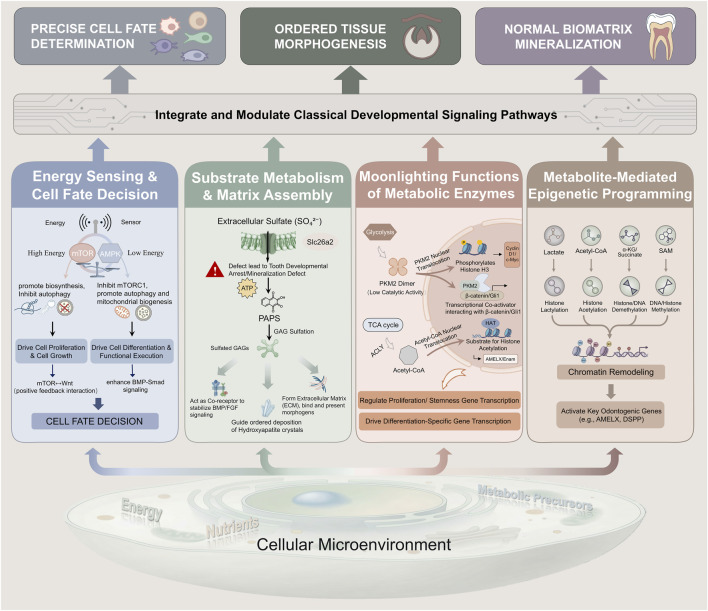
A Metabolic Regulatory Framework for Tooth Development: Integration of Four Core Mechanisms. This integrated schematic illustrates the core paradigm of metabolic activity as a fundamental programming layer for tooth development. The framework consists of four regulatory pillars: 1) Energy sensing and cell fate decision, centered on the AMPK/mTOR signaling hub that senses energy status; 2) Specific substrate metabolism and matrix assembly, exemplified by sulfate metabolism, which provides essential precursors for extracellular matrix assembly and mineralization; 3) Moonlighting signaling functions of metabolic enzymes, such as pyruvate kinase M2 isozyme and ATP citrate lyase, which shuttle between metabolic catalysis and gene transcriptional regulation; 4) Metabolite-mediated epigenetic programming, where key metabolites like acetyl-CoA, α-ketoglutarate, and lactate serve as substrates or signals for histone acetylation, demethylation, and lactylation modifications, respectively. These pillars collectively receive input from the cellular microenvironment and, by deeply coupling with and finely modulating the activity of classical developmental signaling pathways, ultimately drive precise cell fate determination, ordered tissue morphogenesis, and normal biomatrix mineralization in a coordinated manner. Key abbreviations: AMPK (AMP-activated protein kinase); mTOR (mechanistic target of rapamycin); Acetyl-CoA (Acetyl coenzyme A); α-KG (α-ketoglutarate).

While we have delineated four core mechanisms for clarity, they do not operate in isolation during development. Instead, they form a highly integrated, bidirectionally communicating dynamic regulatory network (Figure 1). This interactivity ensures that metabolic information is precisely decoded and translated into morphogenetic instructions.

First, the energy-sensing system acts as the command hub of the network. The AMPK/mTOR hub not only directly determines the balance between anabolism and catabolism but also broadly regulates the activity of key enzymes in the other three mechanisms via post-translational modifications such as phosphorylation. For instance, activated AMPK can phosphorylate and activate ACLY, boosting acetyl-CoA production, thereby directly influencing histone acetylation levels within the “metabolite epigenetic programming” pillar. Conversely, the biosynthetic processes driven by the “enzyme moonlighting” and “substrate metabolism” pillars continuously consume ATP and alter the AMP/ATP ratio, providing real-time feedback on material synthesis status to the AMPK/mTOR energy-sensing center.

Second, metabolites serve as the universal language for inter-mechanism dialogue. For example, the status of both “energy sensing” and “substrate metabolism” collectively determines the cellular abundance of key metabolites such as acetyl-CoA, α-KG, and lactate. These metabolites then act as substrates or signaling molecules for the “metabolite epigenetic programming” pillar, directly reshaping the chromatin landscape. Altered chromatin states, in turn, transcriptionally regulate the expression of enzymes involved in glycolysis, oxidative phosphorylation, or sulfate metabolism, thereby influencing the long-term capacity of “energy sensing” and “substrate metabolism”. Lactate is a paradigmatic example: it is both an end-product of anaerobic glycolysis (linked to energy status) and a precursor for histone lactylation (epigenetic programming), and also functions as an intercellular signaling molecule, perfectly illustrating the central role of metabolites in integrating different levels of regulation.

Finally, the moonlighting functions of metabolic enzymes are critical integration nodes. Enzymes like PKM2 have their activity and subcellular localization influenced by energy status and metabolite concentrations. Once translocated into the nucleus, they can directly regulate transcriptional programs related to proliferation and metabolism, thereby directly coupling cytosolic metabolic states with nuclear gene output.

In summary, the metabolic programming of tooth development is an integrated network orchestrated by the energy-sensing hub, using metabolites as signaling carriers, transduced via the moonlighting functions of metabolic enzymes, and ultimately reliant on specific substrate metabolism for building materials. Understanding the interactive crosstalk within this network is crucial for revealing how metabolism acts as a systemic instructive force, rather than merely a permissive background, to precisely guide the entire developmental process.

## 3 Spatiotemporal dimensions: metabolic reprogramming drives the four key stages of tooth development

Tooth development is a continuous and dynamic process, with each stage having distinct demands for energy, biosynthetic precursors, and specific metabolites. Metabolic reprogramming is not a static background but an active and precisely evolving driving program that progresses with the developmental clock. Through spatiotemporally specific metabolic network regulation, it ensures the orderly progression of morphogenesis, cell differentiation, and tissue mineralization.

### 3.1 Initiation and dental lamina formation: establishing the metabolic foundation and epigenetic landscape

During the initial stage of tooth development, interactions between oral epithelium and cranial neural crest-derived mesenchymal cells lead to the formation of the dental lamina. Cells at this stage are in a relatively primitive and highly proliferative state, and their metabolic characteristics lay the groundwork for subsequent developmental potential.

Hypoxic Microenvironment and HIF Signaling: Early tooth development often occurs within a relatively hypoxic microenvironment. Hypoxia-inducible factor-1α (HIF-1α) is stably expressed during this phase and promotes the expression of glycolysis-related genes including GLUT1 and LDHA through transcriptional regulation, enhancing anaerobic glucose uptake and lactate production (Fang et al., 2023). Meanwhile, HIF-1α forms a positive feedback loop with Wnt/β-catenin signaling, collectively promoting ectodermal differentiation (Shen et al., 2022). This hypoxic metabolic state thus constitutes a crucial regulatory layer in the initiation of tooth development.

Anaerobic Glycolytic Predominance: In early tooth development, cells primarily rely on anaerobic glycolysis to generate ATP and biosynthetic intermediates (Peng et al., 2023; Holomková et al., 2024). This metabolic mode rapidly provides energy and carbon skeletons for nucleic acid and amino acid synthesis, supporting rapid cell proliferation (Lunt and Vander Heiden, 2011). Concurrently, intracellular levels of acetyl-CoA (a key metabolic intermediate and essential substrate for histone acetylation as defined in previous Section 2.3, 2.4) are relatively high, supplying ample substrate for histone acetylation. This helps maintain a relatively open chromatin structure, preserving the developmental pluripotency of cells and keeping target genes of key signaling pathways in a “primed” state for activation (Moussaieff et al., 2015).

Priming of Energy Sensors: Core energy sensors, the AMPK and mTOR pathways, begin to exert regulatory functions at this stage. As detailed in Section 2.1, AMPK and mTOR constitute the upstream hub that senses cellular energy status and directs cell behavior. A favorable nutrient environment activates mTORC1, promoting global protein synthesis and driving initial tooth germ growth. In contrast, mild metabolic stress or specific developmental signals may locally activate AMPK, preparing for early cell fate decisions (Pawar et al., 2021).

Integration of Mechanical Stress, Metabolic Reprogramming, and Developmental Signaling: The initiation and patterning of tooth development are guided not only by classical biochemical signals but also profoundly regulated by the mechanical microenvironment. Emerging research reveals that mechanical stimuli drive specific metabolic reprogramming within cells, which in turn modulates the epigenetic landscape and the activity of signaling pathways, ultimately precisely directing cell fate and tissue morphology. This integrated “mechano-metabolic-signaling” paradigm plays a central role in both tooth development initiation and subsequent adaptive remodeling. At the initiation stage of tooth development, mechanical force is a critical regulatory factor. For instance, studies on miniature pigs and human tooth germs have shown that mechanical stress accumulated in the jawbone prior to deciduous tooth eruption inhibits the initiation of permanent tooth germs via the integrin β1-ERK1-RUNX2 signaling axis. The release of this stress triggers the dental lamina into the morphogenetic program by altering the activity of RUNX2 and Wnt signaling (Wu et al., 2020). This mechanotransduction process relies on basal cellular metabolism to provide energy and biosynthetic support. More directly, metabolic reprogramming has been identified as a core executive step downstream of mechanical signals. Taking orthodontic tooth movement as an example, applied mechanical force can significantly elevate lactate levels in the local microenvironment (Zhai et al., 2022). This force-induced lactate acts as a key metabolic signaling molecule. By driving mitochondrial functional remodeling and epigenetic modifications such as histone lactylation, it directly regulates the expression of genes related to osteogenic differentiation, proliferation, and migration in bone marrow mesenchymal stem cells, thereby driving precise alveolar bone remodeling. In summary, from developmental initiation to adult tissue remodeling, mechanical stimulation initiates specific metabolic reprogramming. This metabolic shift then regulates the activity of core developmental signaling pathways through metabolite signaling and epigenetic mechanisms, ultimately determining cell fate and tissue construction. This integrative framework tightly links the mechanical environment, cellular metabolism, and genetic programs, highlighting the central role of metabolism in sensing and translating physical signals into biological instructions.

### 3.2 Morphogenetic phase (bud to bell stage): metabolic heterogeneity shapes tooth morphology

As the tooth germ progresses through the bud, cap, and bell stages, its morphology gradually becomes defined. During this period, cell populations in different regions of the tooth germ (e.g., the enamel knot, dental papilla tip, cervical loop) exhibit significant metabolic heterogeneity, which directly participates in establishing the early morphological patterns of the tooth germ.

Direct Regulation of Key Signaling Pathways in Tooth Development by Specific Metabolic Substrates and Modifications: In critical signaling centers particularly the enamel knot, cells highly express and secrete morphogens including FGF, BMP, and Shh to drive tooth formation (Novacescu et al., 2025). O-GlcNAcylation is highly active in this region, with its catalytic enzyme OGT specifically and highly expressed in the enamel knot. Its substrate UDP-GlcNAc integrates fluxes from metabolic pathways such as glucose and amino acid metabolism, making O-GlcNAcylation a “metabolic sensor” (Yang and Qian, 2017). Functionally, Inhibiting OGT activity directly disrupts normal enamel knot cell function, impairs the expression of key developmental signals including β-catenin, FGF4, and Shh, and leads to cusp malformation and defects in hard tissue formation (Pokharel et al., 2023). Furthermore, lipid metabolites also participate in signaling regulation: oxysterols, derived from cholesterol metabolism, act as endogenous ligands for the Hedgehog pathway and are enriched in the enamel knot region. By modulating Shh signaling activity, they may further influence cusp patterning (Corcoran and Scott, 2006; Daggubati et al., 2022).

**FIGURE 2:**
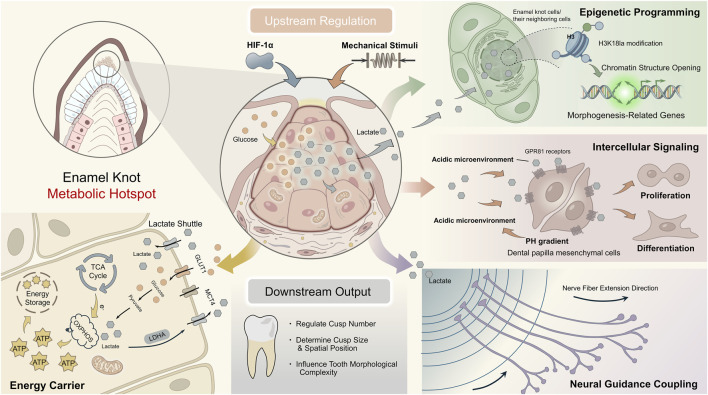
The Central Hub Function of Lactate Signaling in Tooth Morphogenetic Phase. This schematic focuses on the key signaling center during tooth morphogenesis—the enamel knot, detailing the central hub function of the metabolite lactate within it. Robust anaerobic glycolysis in enamel knot epithelial cells generates a large amount of lactate. This lactate executes four key functions: First (Energy Carrier), it can be taken up and metabolized by mesenchymal cells as a metabolic fuel, entering the tricarboxylic acid cycle to provide energy for subsequent energy-demanding processes. Second (Epigenetic programming), as an epigenetic modification substrate, it enters the nucleus and induces lactylation modification at lysine 18 of histone H3, directly opening chromatin and activating the transcription of enamel knot-specific genes. Third (Intercellular Signaling), as a paracrine signaling molecule secreted extracellularly, it activates the GPR81 receptor on the membrane of adjacent dental papilla mesenchymal cells, modulating intracellular signaling pathways to coordinate cell proliferation and differentiation. Fourth (Neural Guidance Coupling), the extracellular concentration gradient it forms can guide the directional growth of sensory nerve endings, participating in the establishment of early innervation patterns. Key abbreviations: H3K18la (Histone H3 lysine 18 lactylation); GPR81 (G-protein coupled receptor 81).

Metabolites as Direct Modulators of Morphogenesis: Metabolic intermediates serve not only as hubs of biochemical reactions but also act directly as substrates for epigenetic modifications, playing a core regulatory role at critical spatiotemporal nodes of tooth morphogenesis. Among these, lactate serves as a paradigm for this mechanism (Figure 2). As introduced in Section 2.4, lactate has been established as a signaling molecule with epigenetic regulatory functions. As the end product of anaerobic glycolysis, lactate is specifically enriched in morphogenetic centers such as the enamel knot and stellate reticulum. Its functions extend far beyond being a metabolic waste product: 1) Energy Carrier: Lactate can be taken up by neighboring cells via the “lactate shuttle,” reconverted to pyruvate by LDHB, and enter the TCA to provide substrate for oxidative phosphorylation after entering the TCA cycle, thereby supporting cell proliferation and biosynthesis (Zhang et al., 2019b) (Figure 2, Energy Carrier); 2) Epigenetic Instruction: Lactate directly induces a novel covalent modification—histone lysine lactylation—thereby opening chromatin structure and directly activating gene programs related to cell proliferation, differentiation, and patterning, ultimately influencing the establishment of cusp number, size, and spatial layout (Liu et al., 2023) (Figure 2, Epigenetic Programming); 3) Intercellular Signaling: Lactate establishes a local concentration gradient, modulates pH, and functions as a microenvironmental signaling molecule to regulate neighboring cell behaviors, such as proliferation and differentiation (Zhang et al., 2019) (Figure 2, Intercellular Signaling); 4) Coupling with Innervation: Studies have revealed that this lactate signal is closely associated with the innervation process, establishing a direct functional link between the metabolic microenvironment and neural patterning (Liu et al., 2023) (Figure 2, Neural Guidance Coupling). Concurrently, other metabolites play significant roles. For example, highly active fatty acid β-oxidation (FAO) in dental papilla cells generates acetyl-CoA (as mentioned earlier), which not only provides substrate for histone acetylation but also, through AMPK sensing of energy status, cooperatively regulates the metabolic switch of cells from proliferation to differentiation, laying the metabolic foundation for subsequent mineralization stages (Xiong et al., 2018; Mei et al., 2025).

In summary, during the morphogenesis stage, the metabolic network integrates deeply with classical developmental signaling pathways through various mechanisms—providing specific substrates, generating direct signaling molecules especially lactate, and shaping the epigenetic landscape. Together, they constitute a spatiotemporally precise instructional system that ultimately determines the number, size, and spatial arrangement of developing tooth morphology.

### 3.3 Cellular differentiation and matrix secretion phase: metabolic specialization meets specific functions

In the late bell stage, ameloblasts and odontoblasts begin their terminal differentiation and commence the massive secretion of extracellular matrix proteins. This stage witnesses a fundamental metabolic reprogramming, shifting from supporting proliferation to enabling large-scale synthesis and secretion (Struys et al., 2005).

Ameloblast Differentiation: During the secretory phase, metabolic reprogramming in ameloblasts provides comprehensive support for the large-scale synthesis and secretion of enamel matrix proteins. Active amino acid metabolism supplies the raw materials for protein synthesis (Elwood and Apostolopoulos, 1975), while efficient mitochondrial oxidative phosphorylation provides the necessary ATP and maintains intracellular calcium ion (Ca^2+^) homeostasis, which is essential for enamel protein secretion and the proper formation of the enamel-dentin junction (Imhof et al., 2020). Concurrently, the cellular metabolic state directly influences protein functional maturation through post-translational modifications. For instance, a specific serine residue (Ser55) in enamelin (ENAM) must undergo phosphorylation by the kinase FAM20C. This ATP-consuming process is a prerequisite for the correct secretion of ENAM, its interaction with other matrix proteins, and ultimately the guided, orderly assembly of enamel. Disruption of this modification directly leads to enamel developmental defects (Yan et al., 2017; Dong et al., 2022). Thus, from raw material supply and energy/ion homeostasis maintenance to precise post-translational modifications, the metabolic network meticulously regulates the synthesis and assembly of the enamel matrix.

Odontoblast Differentiation: The terminal differentiation and functional execution of odontoblasts during the secretory phase are precisely regulated by a multi-dimensional metabolic network. The core objective of this metabolic reprogramming is to support the highly energy- and material-intensive biological processes of collagen matrix synthesis and protein secretion (Meng et al., 2024). A network of key metabolic sensors and regulators finely coordinates the odontogenic process. For example, AMPK, acting as an energy sensor (as mentioned in Section 2.4), promotes mitochondrial metabolism by activating the SIRT1-PGC-1α axis, thereby enhancing odontoblast differentiation and mineralization capacity (Jiang et al., 2019). Conversely, dysfunction within this metabolic network directly leads to cellular impairment. For instance, insufficient ATP synthesis and excessive production of mitochondrial reactive oxygen species (mtROS) resulting from mitochondrial dysfunction can inhibit odontoblast differentiation, activate inflammatory pathways, and ultimately lead to cell death, disrupting dentin formation (Matsuishi et al., 2018; Wu et al., 2022; Tao et al., 2024). Therefore, strategies targeting metabolic homeostasis (e.g., antioxidant interventions) are regarded as novel approaches for maintaining odontoblast function and promoting reparative dentin formation.

### 3.4 Mineralization and maturation phase: the ultimate coordination of ion transport and metabolic homeostasis

Following the deposition of the hard tissue matrix, development enters the mineralization and maturation phase. The core focus of metabolic activity during this stage is to create and maintain a local microenvironment conducive to the nucleation, growth, and maturation of hydroxyapatite crystals, essentially requiring precise cellular regulation of mineral ion metabolism and the local physicochemical environment.

Metabolism and Transport of Mineral Ions: Ameloblasts and odontoblasts not only actively transport calcium ions (Ca^2+^) and inorganic phosphate (PO_4_^3-^) across their membranes via specific transporters but also subject these ions to sophisticated “metabolic processing” intracellularly. Mitochondria, as key metabolic organelles, actively participate in calcium ion storage and buffering (Tang et al., 2020) and may be involved in phosphate metabolism through inner membrane phosphate transporters. This influences local phosphate availability and energy status (e.g., ATP, UTP levels), thereby directly regulating the ionic supersaturation state at the mineralization front and crystal nucleation kinetics (Seifert et al., 2015). Furthermore, the citrate metabolic pathway directly impacts the quality of tooth hard tissue mineralization by regulating the specific deposition of this metabolite within hydroxyapatite crystals, influencing crystal structure and mechanical properties (Dirckx et al., 2022). This finding reveals that the active deposition of specific metabolic intermediates is a key mechanism by which cells precisely control the structure and mechanical performance of biomineralization.

Metabolism-Driven Microenvironment Regulation: During enamel maturation, ameloblasts regulate the pH of the extracellular space through active ion transport and secretory functions. This process critically depends on the cell’s acid-base balance metabolism, particularly the CO_2_ hydration reaction catalyzed by carbonic anhydrase, which generates bicarbonate (HCO_3_^−^) to neutralize acidic metabolic products like lactate (Lacruz et al., 2010). The resulting increase in local pH effectively reduces the activity of mineralization inhibitors such as carbonate ions (CO_3_^2-^), creating the optimal chemical conditions for the oriented growth and final maturation of hydroxyapatite crystals (Li et al., 2023).

Termination and Transition of the Metabolic Program: Upon completion of mineralization, ameloblasts undergo programmed apoptosis (Lacruz et al., 2017; Matalova et al., 2012), and their vigorous anabolic and secretory metabolic activities cease. Odontoblasts transform into quiescent pulp cells (Rajan et al., 2020), switching their metabolic mode from a high-energy-consumption secretory state to a low-energy mode focused on basal maintenance and stress response. This programmatic downscaling and transition of metabolic activity marks the conclusion of the metabolic cycle of tooth development and the establishment of the metabolic homeostasis characteristic of a mature tooth organ.

### 3.5 Summary and perspective: metabolic switching as an active instructional command in developmental programming

**FIGURE 3:**
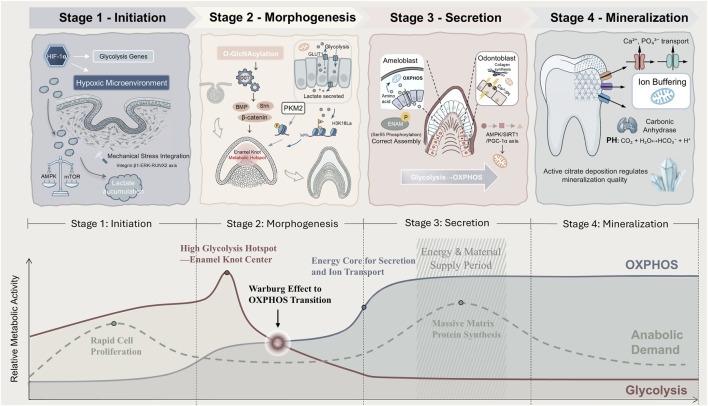
Spatiotemporal Atlas of Metabolic Reprogramming During Tooth Development. This atlas systematically depicts the dynamic reprogramming trajectories of core metabolic pathways throughout tooth development. Following the developmental timeline (left to right), the atlas sequentially covers the initiation, morphogenesis, secretion, and mineralization stages. The bottom curve layer shows that anaerobic glycolytic activity peaks during early development, particularly within the enamel knot region of the morphogenesis stage. Subsequently, a fundamental metabolic switch occurs, with OXPHOS activity surging to become the dominant pathway, supporting the massive energy demands of protein synthesis and ion transport. Anabolic demand exhibits two peaks, corresponding to periods of rapid cell proliferation and extensive matrix secretion, respectively. The top layer integrates the morphological features and key metabolic events of each stage. The core metabolic features of each stage are summarized as follows:1. Initiation Stage: Dominance of HIF-1α-stabilization-driven anaerobic glycolysis; accumulation of glycolytic intermediates. 2. Morphogenesis Stage: Peak levels of lactate, which induces histone lactylation (H3K18la); active O-GlcNAcylation mediated by highly expressed OGT, modulating morphogen signaling. 3. Secretion Stage: Metabolic shift towards OXPHOS accompanied by enhanced mitochondrial biogenesis; elevated nuclear acetyl-CoA supporting histone acetylation and transcriptional burst; crucial post-translational modifications such as enamelin phosphorylation dependent on FAM20C. 4. Mineralization Stage: Carbonic anhydrase-mediated regulation of extracellular pH; specific deposition of citrate within hydroxyapatite crystals; active ion transport metabolism. Key abbreviations: HIF-1α (Hypoxia-Inducible Factor 1-alpha); OXPHOS (Oxidative Phosphorylation).

In summary, the spatiotemporal progression of tooth development is fundamentally a series of precisely sequenced metabolic reprogramming events. From the initial reliance on anaerobic glycolysis to establish the metabolic and epigenetic foundation, through the metabolic heterogeneity and signaling coupling that shapes tooth morphology during morphogenesis, to the metabolic specialization for large-scale matrix synthesis in the secretion phase, and finally to the precise regulation of ionic homeostasis and the microenvironment for tissue maturation during mineralization, the metabolic network consistently acts as the core driving and integrating program. Mechanisms such as energy sensing, metabolite signaling, post-translational modifications, and ion metabolism work in a stage-specific, coordinated manner to translate the cellular nutritional and energy status into precise developmental instructions (Figure 3). However, the field currently lacks a detailed understanding of the precise molecular switches and dynamic regulatory networks that drive these critical transitions. For instance, how do signals from the enamel knot initiate the switch to the secretory metabolic phenotype? How do core hubs like AMPK/mTOR and HIF-1α integrate information and execute commands at the transition nodes? How do transient fluctuations in metabolites “lock in” new cell fates through epigenetic remodeling? Elucidating the dynamic mechanisms of these inter-stage metabolic switches represents the next frontier in understanding the spatiotemporal precision of metabolic programming. In the future, leveraging single-cell multi-omics dynamic tracing, high-resolution live-cell metabolic imaging, and spatiotemporally specific genetic perturbation models holds promise for uncovering the secrets of these “metabolic toggle switches.” This will ultimately enable a leap from describing correlations to establishing regulatory causality, offering novel targets for precisely intervening in developmental timing.

## 4 Metabolic disorders and dental developmental anomalies: a clinical perspective

Tooth development is a process highly sensitive to cellular metabolic status. Any hereditary or acquired disorder affecting energy production, biosynthesis, or metabolic signaling can disrupt the spatiotemporal coordination of this intricate program, leading to structural abnormalities or even developmental arrest. Examining these metabolic disturbances from a clinical perspective not only reveals the etiology of diseases but also inversely confirms the central role of metabolic regulation in tooth development.

### 4.1 Hereditary metabolic diseases: developmental program errors caused by single-gene defects

Specific single-gene mutations can directly disrupt key metabolic pathways essential for tooth development. The resulting phenotypes provide “natural knockout” models for understanding the functions of metabolites.

Hypophosphatasia and Phosphate Metabolism Imbalance: Hypophosphatasia, caused by mutations in the ALPL gene, leads to loss of tissue-nonspecific alkaline phosphatase activity. This enzyme is crucial in osteoblasts and odontoblasts, responsible for hydrolyzing inorganic pyrophosphate (a potent mineralization inhibitor) and releasing inorganic phosphate. Its functional deficiency results in disordered extracellular phosphate metabolism and pyrophosphate accumulation, severely inhibiting hydroxyapatite deposition. Patients present with enamel hypoplasia in both deciduous and permanent teeth, poor dentin mineralization, absence of cementum, along with characteristic root resorption and premature tooth loss (Foster et al., 2017). The specific mechanisms by which hypophosphatasia leads to dental developmental defects extend beyond simple mineral ion imbalance. Research has indicated that the abnormal accumulation of inorganic pyrophosphate (PPi) directly interferes with early tooth germ morphogenesis. PPi can disrupt ECM assembly by inhibiting the function of ANKH (a channel protein that transports intracellular PPi to the extracellular space) and competitively antagonize ATP-mediated activation of P2X/P2Y purinergic receptors, thereby affecting intercellular communication and cell migration (Hessle et al., 2002). More importantly, the deficiency of TNSALP affects the metabolism and differentiation of dental follicle cells, preventing the formation of normal cementum and periodontal ligament attachment. This is the fundamental cause of short root development, cementum aplasia, and premature tooth loss (Foster et al., 2013). Concurrently, PPi accumulation also inhibits the maturation and polarity establishment of ameloblasts and odontoblasts, leading to concurrent impairment in the secretion and mineralization of enamel and dentin matrices (Yadav et al., 2011). Therefore, hypophosphatasia serves as an exemplar of how dental development programs can be disrupted at multiple levels—from the mineralization microenvironment and intercellular signaling to precursor cell differentiation.

Sulfate Transport Disorder and Matrix Assembly Defects: As mentioned previously, sulfate transport disorders caused by mutations in SLC26 genes affect the synthesis of sulfated glycosaminoglycans. In addition to skeletal abnormalities, they are often accompanied by tooth agenesis (congenital absence of teeth), small tooth crowns, and poor mineralization (Yin et al., 2017). The molecular mechanism lies in the fact that sulfated glycosaminoglycans are essential co-receptors for key morphogen signaling such as FGF and BMP. Their deficiency prevents the tooth germ from receiving sufficient developmental instructions, causing arrest at early stages (Yoshida et al., 2024; Härkönen et al., 2021). This highlights the supportive role of specific substrate metabolism for developmental signaling pathways.

Mitochondriopathies and Energy Crisis: Mutations in genes encoding mitochondrial oxidative phosphorylation complex proteins or mtDNA lead to mitochondriopathies characterized by energy deficiency. Although phenotypes vary, many patients exhibit delayed tooth development, enamel hypoplasia, and dentin formation defects. Animal models (e.g., K320E-TwinkleEpi mice) confirm that epithelium-specific mitochondrial functional failure is sufficient to cause ameloblast differentiation collapse and complete absence of enamel matrix secretion (Imhof et al., 2020). This extremely demonstrates the irreplaceable role of mitochondrial oxidative phosphorylation as the “power engine” for development.

### 4.2 Impact of systemic metabolic diseases: systemic imbalances disrupting local development

Systemic metabolic disorders indirectly but broadly affect tooth development by altering the physicochemical properties of the developmental microenvironment and the supply of cellular metabolic substrates.

Maternal and Childhood Diabetes: The hyperglycemic environment exerts multiple negative effects on tooth development. Animal models show that offspring of diabetic dams or diabetic young mice often exhibit delayed tooth eruption, reduced enamel mineralization, and decreased dentin formation (Pascon et al., 2019; Kobayashi et al., 2024). The mechanisms involve: 1) Osmotic diuresis leading to imbalances in amniotic fluid or body fluid electrolytes (e.g., calcium, phosphorus) (Gutowska et al., 2011); 2) Oxidative stress induced by hyperglycemia damaging the function of ameloblasts and odontoblasts (Horsophonphong et al., 2021); 3) Hyperglycemia targeting lineage-specific genes in ameloblast and odontoblast precursors, inhibiting their maturation and differentiation processes, directly impairing the self-renewal and differentiation potential of stem cell pools in the dental pulp/papilla (Hermans et al., 2022); 4) Activation of the TLR4/NF-κB signaling pathway in dental mesenchymal/epithelial cells of T1DM rats, which exacerbates proliferation and apoptosis in these cells while simultaneously inhibiting SMAD1/5/8 phosphorylation, thereby reducing odontoblast differentiation and dentin formation (Chen et al., 2017a; Lyu et al., 2020); 5) Increased DNA methylation in epithelial stem cells due to downregulation of Apex1 leading to upregulation of Dnmt1, resulting in suppressed proliferation and impaired incisor enamel formation (Chen et al., 2017b). This indicates that maintaining systemic glucose and hormonal homeostasis is crucial for normal tooth development.

Malnutrition and Trace Element Deficiencies: Protein-energy malnutrition, deficiencies in vitamins (e.g., A, C, D), and minerals (e.g., calcium, phosphorus, fluoride) have all been reported to be associated with dental developmental defects (Arnold et al., 2021). For example, vitamin D deficiency affects intestinal calcium and phosphorus absorption and bone/tooth mineralization (Mortensen et al., 2022); vitamin C is a cofactor for collagen prolyl hydroxylase, and its deficiency leads to defective collagen synthesis in the dentin matrix (Fujii, 2021). These conditions essentially deprive tooth development of required metabolic raw materials and cofactors, limiting the execution of the developmental program from the supply side.

### 4.3 Environmental and pharmaceutical interference: exogenous metabolic toxicants

In addition to hereditary and systemic disorders, specific environmental chemicals and therapeutic drugs can act as exogenous metabolic disruptors, causing irreversible developmental defects by sabotaging the core metabolic programs of developing tooth cells. These agents directly hijack cellular processes of energy production, biosynthesis, or signal transduction, disrupting the spatiotemporal precision of development at its source.

#### 4.3.1 fluoride exposure: a multi-layered metabolic assault

Excessive fluoride intake (fluorosis) is a classic environmental cause of enamel hypoplasia (dental fluorosis). Its pathological essence is a cascade of metabolic disturbances, from subcellular organelles to gene expression.

Fluoride ions first directly attack the cell’s central energy metabolism, inhibiting mitochondrial respiratory chain complexes to impede OXPHOS, creating an ATP synthesis crisis and forcing cells to shift to inefficient anaerobic glycolysis for energy (Ba et al., 2022). Simultaneously, they severely disrupt the calcium and phosphate homeostasis of ameloblasts, interfering with endoplasmic reticulum calcium stores and mitochondrial phosphate transport, thereby dismantling the ionic gradients and local supersaturation essential for enamel mineralization (Avila-Rojas et al., 2022).

The collapse of energy and ion homeostasis is a key driver of the burst in reactive oxygen species (ROS). The obstructed electron transport chain leads to increased electron leakage and massive production of mitochondrial ROS, such as superoxide anion (O_2_•^-^). Concurrently, enhanced anaerobic glycolysis causes accumulation of the reduced coenzyme NADH, potentially further exacerbating ROS generation through the activation of alternative pathways like NADPH oxidases (Balasubramanian and Perumal, 2022; Zhang et al., 2023). This burst of oxidative stress not only directly damages mitochondrial DNA, lipids, and proteins but also causes abnormal, sustained activation of key signaling proteins, such as MAPK kinases, through oxidative modification (Suzuki et al., 2015).

A more profound impact occurs at the epigenetic level. This metabolic disturbance induces genome-wide DNA methylation pattern disruptions and specific histone modification alterations including H3K9 deacetylation, leading to a more repressive chromatin structure and consequently silencing the transcription of key genes like those for enamel matrix proteins (Pan et al., 2020; Daiwile et al., 2019). Stress pathways such as MAPK/JNK, activated by metabolic pressure (Suzuki et al., 2015), engage in aberrant crosstalk with developmentally crucial signaling pathways that are themselves epigenetically disrupted, ultimately misguiding cell fate (Ma et al., 2020).

This comprehensive metabolic assault also interferes with ameloblast cell cycle progression (e.g., causing G1/S phase arrest) (Hayashi and Tsutsui, 1993), induces non-physiological endoplasmic reticulum stress (ERS) and the unfolded protein response (UPR) (Zhang et al., 2016), resulting in insufficient enamel matrix secretion and premature termination of its maturation and mineralization. Therefore, dental fluorosis is not merely a mineralization defect but a quintessential outcome of an exogenous metabolic toxicant systematically disrupting the integrated cellular network encompassing “energy metabolism–redox balance–ion homeostasis–epigenetics–signal transduction–cell cycle.” This ultimately manifests histologically as enamel hypoplasia and hypomineralization.

#### 4.3.2 Chemotherapeutic drugs: metabolic toxicity to proliferating cells

Cytotoxic chemotherapeutic agents (e.g., alkylating agents, antimetabolites) used in childhood cancer treatment are designed to target rapidly dividing tumor cells but equally impact the actively developing cells of the tooth germ. The primary mechanism involves directly inducing apoptosis in these developing cells by interfering with DNA replication and repair. This process is accompanied by severe oxidative stress and mitochondrial dysfunction (Jiang et al., 2023). The consequence is the depletion of the odontogenic progenitor cell pool and the obstruction of tooth germ morphogenesis, clinically manifested as short-root anomaly (root dysplasia), enamel hypoplasia, tooth eruption disturbances, and multiple dental agenesis (Al-Ansari et al., 2018; Cubukcu et al., 2012). This type of damage highlights the extreme vulnerability of developing cells to metabolic and genotoxic stress, with effects that are broad and non-specific.

**FIGURE 4:**
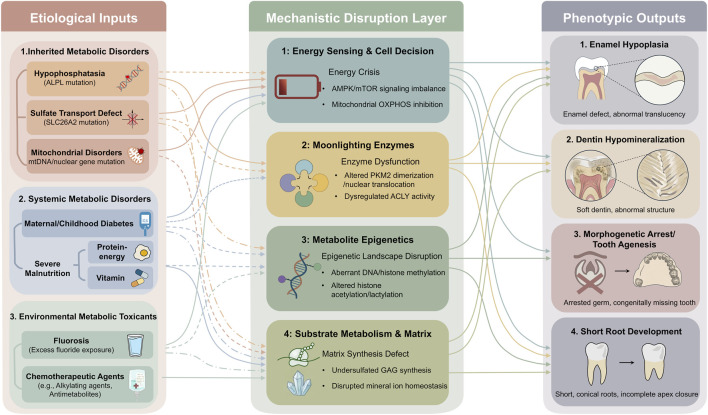
Pathogenic Network of Tooth Developmental Defects Induced by Metabolic Dysregulation. This pathogenic network diagram systematically reveals the shared molecular logic by which hereditary, systemic, and environmental metabolic disorders disrupt the precise program of tooth development. The etiologies listed on the left, including hypophosphatasia caused by ALPL gene mutations, sulfate transport disorder due to SLC26A2 mutations, maternal or childhood diabetes, malnutrition, and fluoride exposure, trigger a series of downstream cascades by targeting and disrupting core metabolic pillars such as energy metabolism, epigenetic modification, and specific substrate synthesis. These cascades encompass cellular energy crisis, reactive oxygen species burst, aberrant genome-wide DNA methylation patterns, impaired synthesis of sulfated glycosaminoglycans, and dysregulation of key developmental signaling pathways. The right side of the network converges on the classic tooth developmental phenotypes eventually resulting from these molecular disturbances, such as enamel hypoplasia, dentin hypomineralization, arrested root development, abnormal crown morphology, and even congenital tooth agenesis. Key abbreviations: ALPL (Alkaline Phosphatase, Biomineralization Associated); SLC26A2 (Solute Carrier Family 26 Member 2).

In summary, the metabolic sensitivity of tooth development makes it a common target for various disorders. Hereditary single-gene diseases disrupt specific substrate metabolism or energy production, precisely truncating the developmental program at the molecular level. Systemic metabolic disorders alter the overall metabolic environment and resource supply, broadly interfering with the developmental microenvironment. Exogenous toxicants directly attack the metabolic core of cells, triggering cascading damage. Although these disturbances originate differently, they ultimately converge on the disruption of the cellular “energy-biosynthesis-signal transduction” network, leading to a spectrum of phenotypes ranging from enamel hypoplasia to root formation defects. Therefore, clinical dental developmental anomalies can be viewed as the “final phenotypic readout” of failed metabolic regulation. A deep understanding of their metabolic etiology not only provides a basis for disease diagnosis and genetic counseling but also points the way toward developing targeted metabolic intervention strategies (Figure 4).

## 5 Frontiers in technology and translational prospects

The deepening understanding of the metabolic basis of tooth development is now being translated into revolutionary research tools and clinical strategies through emerging technologies, propelling dental medicine into a new era of precise regulation and regenerative therapy.

### 5.1 Multi-omics integration and spatiotemporal resolution technologies

**FIGURE 5:**
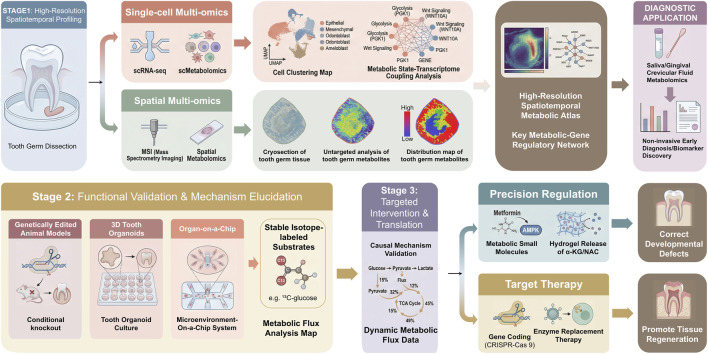
Advanced Technological Roadmap for Deciphering and Intervening in Tooth Developmental Metabolic Programming. This technological roadmap outlines an interdisciplinary research strategy, spanning from basic mechanistic deciphering to clinical translational applications, targeting tooth developmental metabolic programming. The route encompasses three progressive stages: The first stage is dedicated to high-resolution spatiotemporal profiling, integrating single-cell RNA sequencing with single-cell metabolomics to reveal cellular heterogeneity, combining spatial metabolomics and mass spectrometry imaging to visualize metabolite distribution *in situ* within tissues, and employing artificial intelligence algorithms to construct predictive metabolism-epigenetics-signaling network models. The second stage focuses on mechanistic validation and dynamic modeling. Utilizing stable isotope tracing for dynamic metabolic flux analysis in models such as conditional gene knockout animals, Microenvironment-On-a-Chip System and three-dimensional tooth organoids validates regulatory nodes within controlled systems. The third stage concentrates on targeted intervention and translation, developing precise regulation strategies based on metabolic small-molecule drugs or metabolically engineered biomaterials, exploring gene editing or enzyme replacement therapy for hereditary metabolic enzymes, and advancing the diagnostic application of salivary metabolomics in early developmental risk screening. Key abbreviations: scRNA-seq (single-cell RNA sequencing); MSI (Mass Spectrometry Imaging).

The integration of single-cell and spatial multi-omics technologies is deciphering the spatiotemporal metabolic atlas of tooth germ development with unprecedented resolution. The combination of single-cell RNA sequencing (scRNA-seq) and single-cell metabolomics (scMetabolomics) can reveal the coupling between metabolic states and transcriptional programs at the individual cell level, precisely identifying key metabolic subpopulations that drive cusp morphogenesis or cell differentiation (Zhang et al., 2025; Caetano et al., 2022). Spatial metabolomics technologies, such as mass spectrometry imaging (MSI), can directly visualize the distribution gradients of metabolites (e.g., lactate, citrate, specific lipids) on tissue sections, providing intuitive evidence of how metabolic heterogeneity corresponds to morphological structures and offering direct proof for the concept of metabolite gradients guiding pattern formation (Dekker et al., 2023; Sasano et al., 2023). Integrating these multi-omics datasets with artificial intelligence holds promise for constructing a dynamic “metabolism-epigenetics-signaling” network model of tooth development, enabling a leap from describing correlations to predicting regulatory relationships (Figure 5, Stage 1).

### 5.2 *In Vitro* model systems and dynamic mechanism validation

Multi-omics analyses have revealed abundant correlations, yet elucidating definitive causal mechanisms requires functional validation models. New-generation *in vitro* model systems provide a powerful platform for this purpose. Genetically edited animal models like conditional knockout mice enable the specific disruption or activation of particular metabolic genes *in vivo*, serving as the gold standard for validating target necessity (Yang et al., 2018). More precise manipulations can be achieved *in vitro*: three-dimensional tooth organoids can highly recapitulate the cellular composition and spatial architecture of developing tooth germs, allowing researchers to meticulously control culture conditions such as oxygen tension, nutrient supply, and metabolite gradients (Shoushrah et al., 2021). Furthermore, organ-on-a-chip systems can incorporate fluid flow control and mechanical loading to dynamically simulate the *in vivo* microenvironment (Mitsiadis and Pagella, 2025). Within these controllable systems, coupling with stable isotope-labeled metabolic flux analysis enables the real-time, quantitative tracing of the metabolic fate of key substrates like glucose and glutamine, generating metabolic flux maps (Wang et al., 2020). This approach directly tests whether specific metabolic reprogramming events drive cell fate transitions. Collectively, these technologies advance metabolic research from static “observation” to dynamic “causal verification”(Figure 5, Stage 2).

### 5.3 Metabolic intervention and precision medicine strategies

Targeted intervention strategies against identified metabolic vulnerabilities or regulatory nodes offer novel pathways for preventing dental developmental anomalies and promoting regeneration (Figure 5, Stage 3).

Metabolic Reprogramming-Guided Tissue Engineering: Mimicking the metabolic microenvironment of tooth germ development *in vitro* using biomaterials or small molecules can precisely control stem cell fate. For instance, employing specific pharmacological modulators such as the AMPK activator metformin or mTOR inhibitors to simulate a state of relative energy restriction may promote the differentiation of dental stem cells into functional cells (Liu et al., 2024; Xu et al., 2021). Directing the epigenetic state of cells by modulating metabolite concentrations like α-ketoglutarate and acetyl-CoA in the culture medium can optimize their odontogenic potential (Wu et al., 2025; Toan et al., 2021).

Therapy Targeting Pathogenic Metabolic Pathways: For hereditary enamel hypoplasia caused by defects in specific metabolic enzymes (Naniwa et al., 2023), gene editing or enzyme replacement therapy represent future exploratory directions (Dong et al., 2022). For acquired conditions like fluorosis, interventions targeting downstream effects such as oxidative stress (using antioxidants like NAC) or epigenetic dysregulation (using histone deacetylase inhibitors) may serve as auxiliary strategies to mitigate pathological damage (Zhang et al., 2023; Yamashita et al., 2024).

### 5.4 Early warning and diagnosis based on metabolic biomarkers

Metabolomic analysis of oral fluids like saliva and gingival crevicular fluid offers the potential for non-invasive, dynamic monitoring of tooth developmental status or risk (Baima et al., 2021). By identifying characteristic metabolic fingerprints associated with specific developmental stages or abnormalities particularly enamel hypomineralization, it may be possible to establish metabolic panels in the future for screening newborns or children at risk for dental developmental issues (Tsagkari et al., 2022). This is significant for the early detection of dental problems caused by systemic metabolic diseases (e.g., diabetes, malnutrition) or occult hereditary disorders, enabling timely intervention.

The frontier of tooth developmental metabolism research is transitioning from mechanistic elucidation toward precise regulation and clinical application. Through single-cell and spatial multi-omics technologies, we can now map metabolic landscapes with high spatiotemporal resolution, while artificial intelligence facilitates the construction of dynamic “metabolism-epigenetics-signaling” network models, enabling a shift from correlative analysis to predictive regulation. Building on these insights, strategies such as small-molecule interventions, biomaterial-based simulation, and gene editing targeting key metabolic nodes offer novel avenues for correcting developmental defects and promoting tooth regeneration. Meanwhile, non-invasive biomarker detection based on oral fluid metabolomics holds promise for early screening and risk warning of developmental anomalies. Together, these advances are driving stomatology into a new era of precision prevention, diagnosis, and therapy grounded in metabolic programming (Figure 5).

## 6 Conclusion

This review systematically delineates the central role of metabolic regulation in tooth development. Research demonstrates that metabolism is far more than just an energy supplier for life processes; it has emerged as the core programming language directing tooth development. From energy sensing and mechanical signal integration in the initiation stage, through the shaping of cusp patterns by metabolic heterogeneity during morphogenesis, to the specialized metabolic support for matrix synthesis and mineralization in the secretion phase, metabolic reprogramming is a continuous thread. Through four core mechanisms—energy/nutrient sensing, moonlighting functions of metabolic enzymes, metabolite-signal-mediated epigenetic regulation, and substrate-specific metabolism—it deeply couples with and instructs classic developmental signaling pathways like Wnt, BMP, and FGF, ensuring spatiotemporal precision. The various dental developmental anomalies resulting from hereditary or acquired metabolic disorders underscore, from the opposite perspective, the critical importance of this network.

Despite significant progress, the field still faces several challenges: (1) Dynamic Network Analysis: Current studies often provide static snapshots or views at specific time points. Future work needs to employ technologies like live-cell imaging and stable isotope dynamic tracing to analyze, *in vivo* and in real-time, the patterns of dynamic changes in metabolic fluxes throughout development, aiming to construct truly dynamic network models. (2) The Mechanism of “Bidirectional Dialogue”: While it is known that metabolism regulates signaling pathways, the molecular details of how signaling pathways, in turn, finely tune the activity of specific metabolic enzymes or metabolic compartmentalization—this “bidirectional dialogue”—remain poorly understood. (3) The Gap from Mechanism to Clinical Translation: The safety, specificity, and efficacy in the complex human environment of many metabolic intervention strategies proven effective in cellular or animal models require rigorous evaluation. Translating metabolism-based regenerative strategies into safe, controllable clinical therapies represents the foremost challenge. (4) A Systems Integration Perspective: Tooth development does not occur in isolation but is influenced by systemic metabolic status (e.g., maternal nutrition, hormone levels). Future research needs to more systematically investigate the interaction between local oral metabolism and whole-body metabolism. This will provide a scientific basis for the “whole-life-cycle health” concept of promoting oral development by regulating systemic health.
